# Overall Survival with [^177^Lu]Lu-PSMA-617 Versus [^177^Lu]Lu-PSMA I&T: A Propensity Score–Matched Real-World Analysis

**DOI:** 10.2967/jnumed.126.272415

**Published:** 2026-09

**Authors:** Natalie Hasenauer, Charlotte Probst, Wiebke Schloetelburg, Tim Jedamzik, Marieke Heinrich, Nicole Zindler, Andreas K. Buck, Anna Katharina Seitz, Philipp E. Hartrampf

**Affiliations:** 1 Department of Nuclear Medicine, University Hospital Wuerzburg, Wuerzburg, Germany; and; 2 Department of Urology, University Hospital Wuerzburg, Wuerzburg, Germany

**Keywords:** Lu-PSMA, PSMA I&T, PSMA-617, mCRPC, radiopharmaceutical therapy

## Abstract

In this study, we compared clinical outcomes and safety profiles of radiopharmaceutical therapy with [^177^Lu]Lu-PSMA-617 and [^177^Lu]Lu-PSMA I&T in patients with metastatic castration-resistant prostate cancer under real-world conditions. **Methods:** This single-center retrospective matched-pair analysis included 140 chemotherapy-pretreated patients with metastatic castration-resistant prostate cancer treated with commercially available [^177^Lu]Lu-PSMA-617 (7.4 GBq every 6 wk) (*n* = 70) or in-house produced [^177^Lu]Lu-PSMA I&T (6.0 GBq every 8 wk) (*n* = 70). Propensity score matching was performed on the basis of age, baseline prostate-specific antigen (PSA), and PSA-positive tumor volume and stratified by Gleason score. Overall survival (OS), biochemical response, prognostic factors, and treatment-related toxicity were analyzed. **Results:** Median OS was comparable between matched cohorts ([^177^Lu]Lu-PSMA I&T: 14 mo; range, 9–16 mo; [^177^Lu]Lu-PSMA-617: 15 mo; range, 10–21 mo); hazard ratio, 1.18; 95% CI, 0.79–1.78; *P* = 0.42). Early PSA response was strongly associated with prolonged OS in both cohorts (*P* < 0.01). Patients treated with [^177^Lu]Lu-PSMA-617 demonstrated a greater median PSA decline (−58% vs. −14%, *P* = 0.02) and higher biochemical response rates (70% vs. 54%). Although quantitative hematologic changes were more pronounced with [^177^Lu]Lu-PSMA-617, Common Terminology Criteria for Adverse Events–graded toxicity remained comparable between cohorts. Multivariable analysis revealed that higher baseline levels of lactate dehydrogenase were significantly associated with shorter OS in both cohorts (hazard ratio, 1.03–1.04 per 10 U/L, *P* < 0.01). **Conclusion:** Treatment with [^177^Lu]Lu-PSMA I&T and [^177^Lu]Lu-PSMA-617 resulted in comparable survival outcomes despite different treatment protocols. Greater biochemical responses and subclinical hematologic changes with [^177^Lu]Lu-PSMA-617 may reflect higher cumulative radiation exposure, although patient-specific dosimetry data were not available to confirm this hypothesis.

## Visual Abstract

**Figure:**
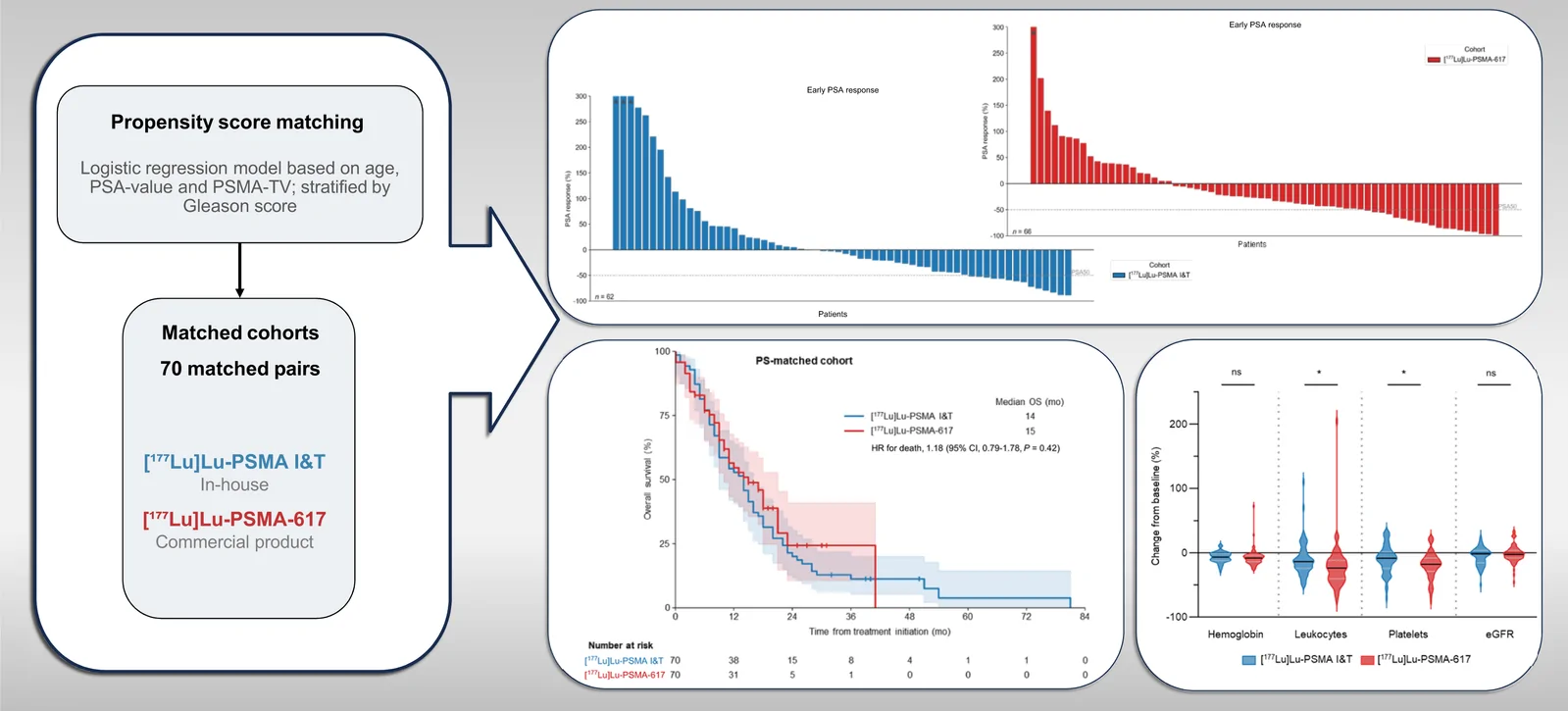

Radiopharmaceutical therapy (RPT) targeting prostate-specific membrane antigen (PSMA) with ^177^Lu has become an established treatment option for patients with advanced metastatic castration-resistant prostate cancer (mCRPC) (1*–*6). The pivotal, prospective, randomized VISION trial established the efficacy of [^177^Lu]Lu-PSMA-617 in heavily pretreated patients, demonstrating improved median overall survival (OS), as well as superior radiographic progression-free survival (rPFS) compared with standard-of-care treatment alone (2). Based on these results, [^177^Lu]Lu-PSMA-617 received approval from the Food and Drug Administration for the treatment of progressive mCRPC after taxane-based chemotherapy and androgen receptor pathway inhibitor therapy. More recently, the PSMAfore trial extended these findings to taxane-naïve patients, showing an rPFS benefit compared with switching to another androgen receptor pathway inhibitor (median, 11.6 mo vs. 5.6 mo; hazard ratio [HR], 0.49) (7).

Although [^177^Lu]Lu-PSMA-617 represents the only commercially available and regulatory-approved PSMA RPT, [^177^Lu]Lu-PSMA I&T has been widely used in institutional settings, with comparable antitumor activity reported (1*,*^4^*,*^8^). Although both compounds belong to a class of urea-binding small molecules, they differ in linker composition and chelators (9*,*^10^).

The recently presented SPLASH trial evaluated [^177^Lu]Lu-PSMA I&T in taxane-naïve patients with mCRPC, similar to the PSMAfore cohort (11). Although the SPLASH trial demonstrated a significant rPFS benefit (median, 9.5 mo vs. 6.0 mo; HR, 0.71), the magnitude of benefit was notably smaller than that observed with [^177^Lu]Lu-PSMA-617 in PSMAfore. This raises the question of whether the apparent difference in efficacy between trials reflects inherent ligand properties or are primarily driven by differences in treatment intensity (dose and schedule effects). Notably, the PSMAfore trial administered 7.4 GBq of [^177^Lu]Lu-PSMA-617 every 6 wk for up to 6 cycles, whereas the SPLASH trial used 6.8 GBq of [^177^Lu]Lu-PSMA I&T every 8 wk for up to 4 cycles.

After regulatory approval of [^177^Lu]Lu-PSMA-617 (Pluvicto; Novartis) in December 2022, our institution transitioned from in-house produced [^177^Lu]Lu-PSMA I&T to [^177^Lu]Lu-PSMA-617, adopting the approved dosing protocol. This transition provided the opportunity to collect real-world comparative data for both compounds under their respective treatment paradigms. Such comparative data may inform evidence-based treatment decisions and protocol optimization across different PSMA ligands.

In this study, we compared the clinical outcomes, prognostic factors, and safety profiles of [^177^Lu]Lu-PSMA-617 and [^177^Lu]Lu-PSMA I&T in a single-center cohort under real-world conditions. We hypothesized that the higher cumulative administered activity of the [^177^Lu]Lu-PSMA-617 protocol would confer a survival benefit and superior biochemical response rates at the cost of greater hematotoxicity and nephrotoxicity.

## MATERIAL AND METHODS

### Patient Cohort

**FIGURE 1.**
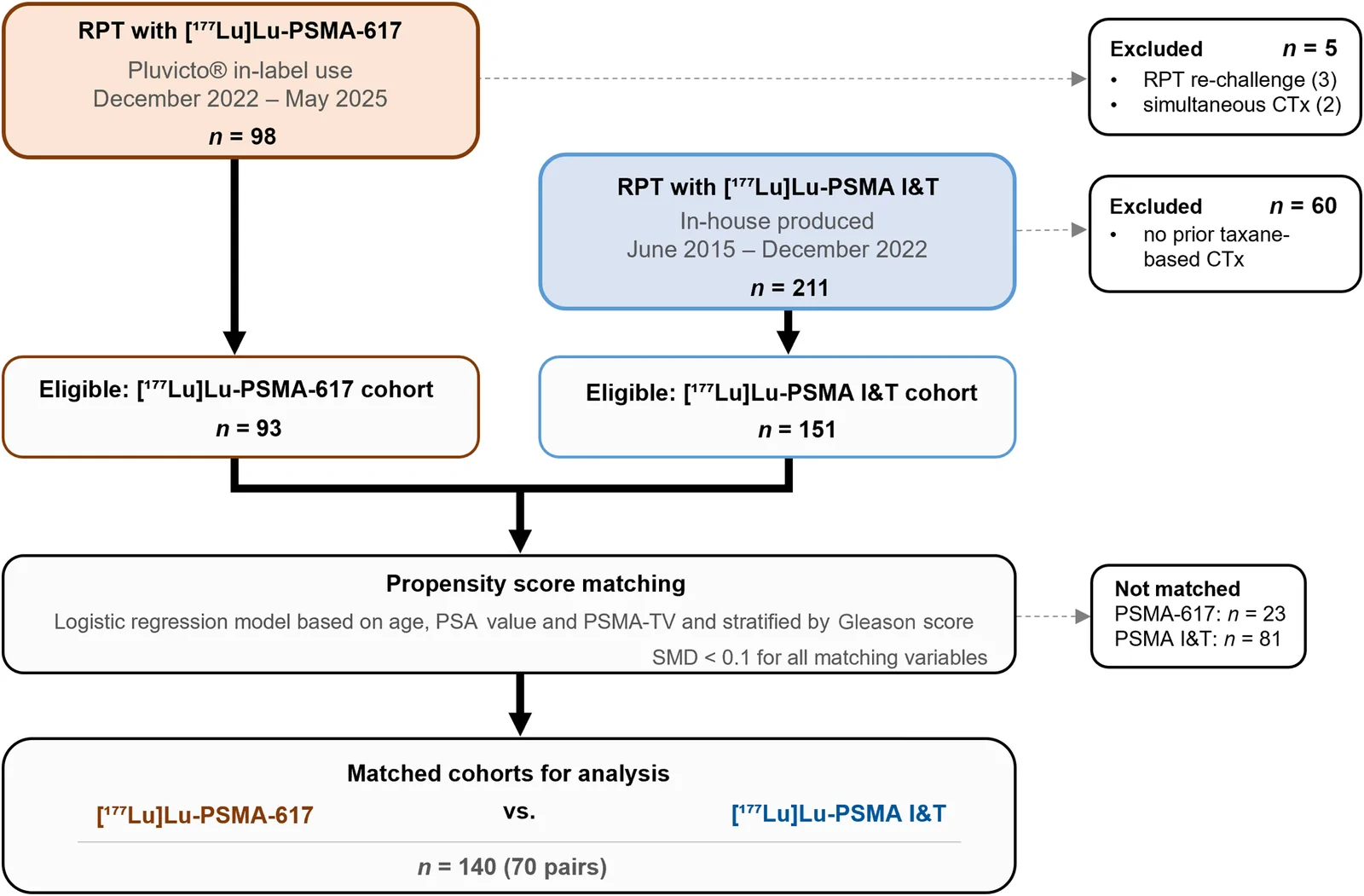
Patient assessment and selection flowchart. CTx = chemotherapy; SMD = standardized mean difference.

This retrospective single-center study included all consecutive patients with mCRPC who received [^177^Lu]Lu-PSMA-617 at our institution between December 2022 and May 2025 (*n* = 98). Of these, 5 were excluded because of simultaneous chemotherapy or RPT rechallenge, resulting in 93 evaluable patients. Propensity score matching was performed to identify a corresponding cohort from patients treated with [^177^Lu]Lu-PSMA I&T between June 2015 and December 2022 (*n* = 211). From this cohort, 60 patients were excluded because they did not receive prior taxane-based chemotherapy, leaving 151 patients available for matching (Fig. 1).

This study was conducted in accordance with the guidelines of the Declaration of Helsinki. Ethical review and approval were waived for this study by the local Ethics Committee because of the retrospective nature of the study (waiver 2026-25-dvhd).

### Pretherapy Work-Up and PET Analysis

All patients received PSMA-targeted PET/CT before RPT. Further information on PET image analysis is provided in the supplemental materials, available at http://jnm.snmjournals.org. Blood samples were taken on the day of admission of the first therapy cycle, including routine hematology (leukocytes, hemoglobin, and platelets) and serum chemistry (serum creatinine, estimated glomerular filtration rate, C-reactive protein, alkaline phosphatase [ALP], aspartate transaminase, lactate dehydrogenase [LDH], γ-glutamyltransferase, and prostate-specific antigen [PSA]). Further details on the laboratory methods, imaging procedures, and radiotracer synthesis are provided in the supplemental materials (12*–*16).

### Treatment Protocol

[^177^Lu]Lu-PSMA I&T was synthesized in-house (synthesis details in the supplemental materials); [^177^Lu]Lu-PSMA-617 was commercially procured. The standard treatment course for [^177^Lu]Lu-PSMA I&T was 6 GBq every 8 wk for 4 cycles, compared with 7.4 GBq of [^177^Lu]Lu-PSMA-617 every 6 wk for 6 cycles per manufacturer’s specifications (17). Therapy continuation beyond 4 or 6 cycles was guided by residual tumor burden and the patient’s overall condition. Early treatment discontinuation was most commonly driven by disease progression, deterioration in performance status, or death, with each decision made by an interdisciplinary tumor board on an individual basis. The predominant indication for dose reductions was renal function impairment (Supplemental Tables 1–3). As part of the standard supportive regimen, all patients received 2000 mL of a balanced electrolyte solution (Sterofundin; B. Braun).

### Toxicity Assessment

Hematologic and renal parameters were monitored each day of hospitalization and were retrospectively reclassified using Common Terminology Criteria for Adverse Events (CTCAE) 6.0 to ensure uniform toxicity reporting (18). As parameters were exclusively assessed on treatment days, missing values are a result of early treatment discontinuation.

### Statistical Analysis

Statistical analyses were performed using Prism version 10 (GraphPad Software) and Python version 3.11.9. Values are presented as medians and ranges. Propensity scores were calculated on the basis of age, log-transformed PSA levels, and log-transformed PSMA-positive tumor volume (PSMA-TV). Univariable and multivariable regression analyses were performed using Cox proportional hazards regression models. Results were reported as HRs with corresponding 95% CIs. Survival curves were estimated using the Kaplan–Meier method and compared via log-rank test. OS was defined as the time from initiation of RPT to death. Early PSA response was defined as any PSA decrease at cycle 2 day 1, with the best PSA response defined as the maximum decrease during treatment. Late PSA response was defined as any decrease in PSA from baseline after the initial increase. For all analyses, a *P* value of less than 0.05 was considered statistically significant. Further information on statistical methods is provided in the supplemental materials.

## RESULTS

### Patient Characteristics After Propensity Score Matching

Before matching, the unmatched cohorts showed significant baseline differences. Most importantly, the [^177^Lu]Lu-PSMA I&T group demonstrated a larger median PSMA-TV (471 mL [range, 9.54–3820 mL] vs. 279 mL [range, 12.3–3146 mL]), a significantly higher rate of liver metastases (34/151 vs. 8/93, *P* = 0.03), and a higher median baseline LDH level (270 U/L [range, 118–1800 U/L] vs. 227 U/L [range, 125–1470 U/L]; *P* = 0.03) compared with the [^177^Lu]Lu-PSMA-617 cohort (Supplemental Table 4; Supplemental Fig. 1).

Propensity score matching yielded 70 well-balanced matched pairs (standardized mean difference of <0.1 for all matching variables). The median age was 74 y (range, 46–86 y) in the [^177^Lu]Lu-PSMA I&T cohort and 71 y (range, 54–84 y) in the [^177^Lu]Lu-PSMA-617 cohort. The median baseline PSA level was 98 ng/mL (range, 4.8–3210 ng/mL) in the [^177^Lu]Lu-PSMA I&T cohort and 83.2 ng/mL (range, 1.1–3150 ng/mL) in the [^177^Lu]Lu-PSMA-617 cohort. The median PSMA-TV was 327 mL (range, 10–3619 mL) in the [^177^Lu]Lu-PSMA I&T group and 246 mL (range, 12–2171 mL) in the [^177^Lu]Lu-PSMA-617 group. Prior treatment history, metastases distribution, and laboratory parameters were well-balanced between groups (Table 1). Only treatment paradigm–related parameters (number of cycles, activity per cycle, cumulative activity) remained different between groups, corresponding with their respective treatment protocols.

**TABLE 1.** Patient Characteristics

Characteristic	[^177^Lu]Lu-PSMA I&T*	[^177^Lu]Lu-PSMA-617*	|SMD|	*P*
Matching variables				
Age at first cycle of RPT (y)	74 (46–86)	71 (54–84)	0.098	
Gleason score			0.00	
7	19 (27)	19 (27)		
8	15 (21)	15 (21)		
9	33 (47)	33 (47)		
10	3 (4)	3 (4)		
PSMA-TV at baseline (mL)	327 (10–3619)	246 (12–2171)	0.002	
Baseline PSA (ng/mL)	98 (4.8–3210)	83.2 (1.1–3150)	0.025	
Clinical variables				
Age at first diagnosis (y)	64.5 (42–84)	64.4 (46–80)		0.99
Time from ID to first cycle of RPT (mo)	82 (16–236)	63 (7–228)		0.68
No. of cycles	3 (1–8)	4 (1–9)		*0.03*
Cumulative activity (GBq)	17.7 (4.8–50.6)	29.5 (7.2–66.3)		*<0.01*
Activity per cycle (GBq)/patient	6.2 (4.0–6.6)	7.3 (6.2–7.7)		*<0.01*
ECOG performance status				
≤1	66 (94)	64 (91)		0.99
≥2	4 (6)	6 (9)		0.99
Metastases				
Bone	66 (94)	64 (91)		0.99
Lymph node	51 (73)	49 (70)		0.99
Liver	11 (16)	7 (10)		0.76
Lung	8 (11)	7 (10)		1.00
Adrenal	2 (3)	3 (4)		1.00
Other	6 (9)	2 (3)		0.76
Prior treatments				
Prostatectomy	36 (51)	30 (43)		0.79
Initial radiotherapy	3 (4)	8 (11)		0.76
Adjuvant or salvage RT	26 (37)	24 (34)		0.99
ADT	70 (100)	70(100)		NA
ARPI	68 (97)	70 (100)		NA
Chemotherapy	70 (100)	70 (100)		NA
Concurrent therapies				
ARPI	15 (21)	22 (31)		0.68
Olaparib	0 (0)	3 (4)		NA
RT	0 (0)	1 (1)		NA
Surgical resection	0 (0)	1 (1)		NA
Baseline laboratory values				
Leukocytes (×10³/µL)	6.5 (2.0–72)	5.9 (2.4–14.1)		0.76
Hemoglobin (g/dL)	12.0 (7.4–15.0)	12.1 (5.4–16.1)		0.76
Platelets (×10³/µL)	234 (91–590)	242 (85–494)		0.99
eGFR (mL/min)	81.5 (36–130)	83 (31–118)		0.76
Creatinine (mg/dL)	0.93 (0.52–1.75)	0.88 (0.51–2.11)		0.69
LDH (U/L)	253 (118–1479)	229 (125–1473)		0.99
CRP (mg/dL)	0.37 (0–11.6)	0.5 (0–17.5)		0.25
AST (U/L)	26.0 (7–163)	26.8 (12.9–449)		0.99
GGT (U/L)	27 (9.2–628)	30 (6.8–1344)		0.58
AP (U/L)	113 (40–1510)	114 (51–2680)		0.91

*n = 70 per group.

SMD = standardized mean difference; ID = initial diagnosis; ECOG = Eastern Cooperative Oncology Group; RT = radiotherapy; ADT = androgen deprivation therapy; NA = not applicable; ARPI = androgen receptor pathway inhibitor; eGFR = estimated glomerular filtration rate; CRP = C-reactive protein; AST = aspartate transaminase; GGT = γ-glutamyltransferase; AP = alkaline phosphatase.

Quantitative data are number and percentage; continuous data are median and range.

### Biochemical Response Between Cohorts

**FIGURE 2.**
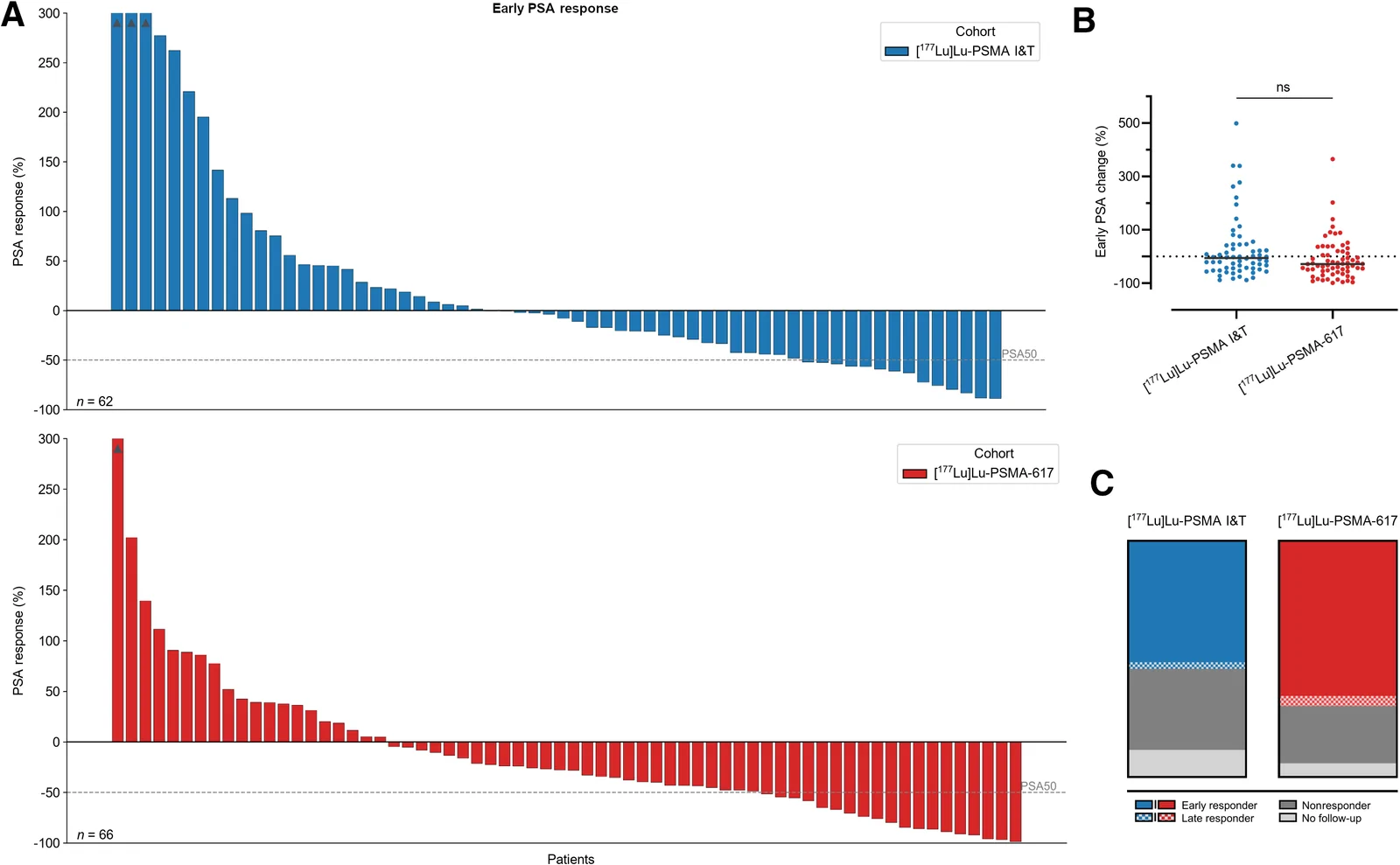
(A) Waterfall plots and (B) scattered-dot plot for early biochemical response as PSA change from baseline for [^177^Lu]Lu-PSMA I&T and [^177^Lu]Lu-PSMA-617. (C) Biochemical response rates stratified by early responders, late responders, and nonresponders, showing low rate of late responders. ns = not significant.

Patients treated with [^177^Lu]Lu-PSMA-617 showed greater declines in PSA levels compared with the [^177^Lu]Lu-PSMA I&T group. The early median change in PSA level was −28% (range, −99% to 365%) versus −5.8% (−89% to 499%), respectively, narrowly missing statistical significance (*P* = 0.052; median difference, −18.95%) (Figs. 2A and 2B). The best PSA response differed significantly between groups, with a median decline of −58% (range, −100% to 365%) in the [^177^Lu]Lu-PSMA-617 group and −14% (−100% to 499%) in the [^177^Lu]Lu-PSMA I&T cohort (*P* = 0.02; median of differences: −27%, Supplemental Fig. 2).

Total PSA response rates were 70% (49/70) for the [^177^Lu]Lu-PSMA-617 cohort and 54% (38/70) for the [^177^Lu]Lu-PSMA I&T group. Early PSA response occurred in 66% (46/70) and 51% (36/70) of patients in the [^177^Lu]Lu-PSMA-617 and [^177^Lu]Lu-PSMA I&T cohorts, respectively, with a decrease of over 50% in PSA levels achieved in 41% versus 39% of patients, respectively. Delayed responses were rare (3 in the [^177^Lu]Lu-PSMA-617 cohort and 2 in the [^177^Lu]Lu-PSMA I&T cohort). PSA progression was observed in 17 patients (24%) in the [^177^Lu]Lu-PSMA-617 cohort and 24 patients (34%) in the [^177^Lu]Lu-PSMA I&T cohort, with follow-up data unavailable in 6% versus 11% of patients in the respective groups (Fig. 2C).

### OS in Matched Cohorts

**FIGURE 3.**
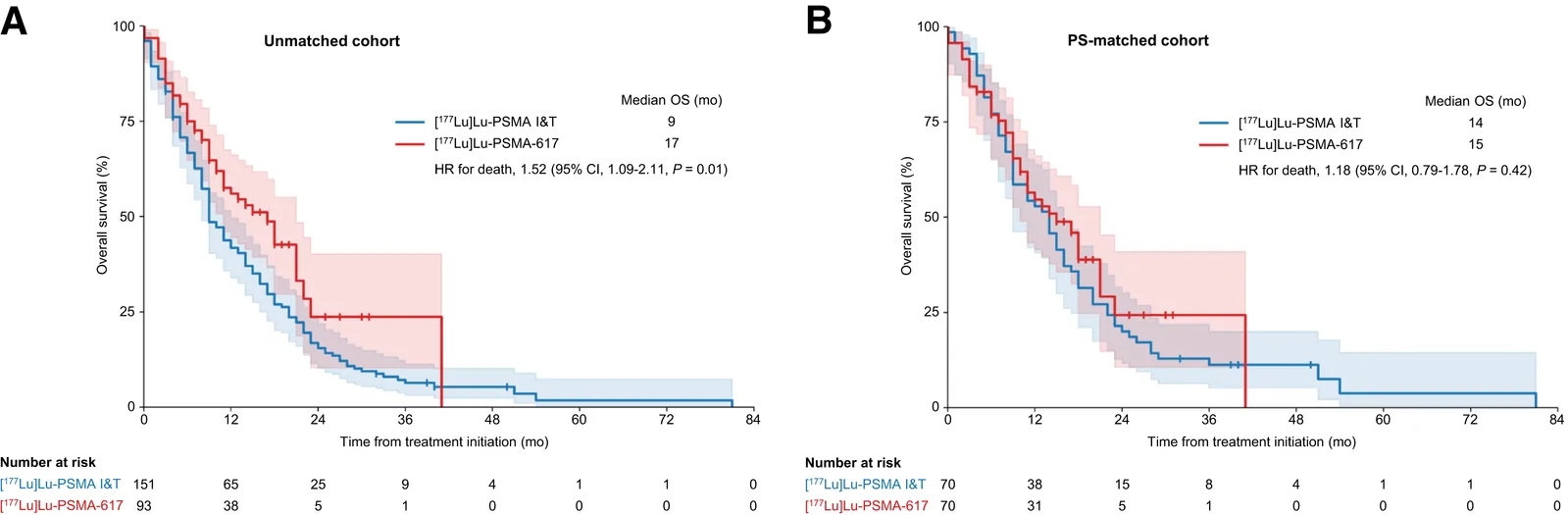
Kaplan–Meier curves for OS from time of treatment initiation in unmatched (A) and propensity score (PS)–matched (B) cohorts.

The median follow-up time differed between cohorts, reflecting the timeline of treatment implementation (50 mo vs. 17 mo in the [^177^Lu]Lu-PSMA I&T and [^177^Lu]Lu-PSMA-617 cohorts, respectively), with fewer deaths recorded in the [^177^Lu]Lu-PSMA-617 cohort (*n* = 39, 56%) compared with the [^177^Lu]Lu-PSMA I&T cohort (*n* = 65, 93%). Unadjusted survival analysis before matching showed a marked difference in OS between groups, with patients treated with [^177^Lu]Lu-PSMA I&T having a significantly shorter median OS compared with those treated with [^177^Lu]Lu-PSMA-617 (9 mo vs. 17 mo; HR, 1.52; 95% CI, 1.09–2.11; *P* = 0.01) (Fig. 3A). However, after propensity score matching, the survival curves converged closely (Fig. 3B). The median OS was 14 mo (range, 9–16 mo) for the [^177^Lu]Lu-PSMA I&T cohort and 15 mo (range, 10–21 mo) for the [^177^Lu]Lu-PSMA-617 cohort, with no statistically significant difference between matched cohorts (HR, 1.18; 95% CI, 0.79–1.78; *P* = 0.42).

### Prognostic Relevance of Baseline Laboratory Values

Univariable Cox regression identified higher baseline levels of C-reactive protein, LDH, ALP, aspartate transaminase, γ-glutamyltransferase, PSA, and PSMA-TV as significantly associated with shorter OS in both groups. Additionally, lower platelet counts in the [^177^Lu]Lu-PSMA I&T cohort and lower hemoglobin levels in the [^177^Lu]Lu-PSMA-617 cohort were significantly associated with shorter OS. Multivariable Cox regression models for C-reactive protein, LDH, ALP, and PSMA-TV were applied for each treatment group. In both cohorts, only higher LDH levels remained prognostic for shorter OS ([^177^Lu]Lu-PSMA I&T: HR, 1.03 per 10 U/L; 95% CI, 1.01–1.05 per 10 U/L; [^177^Lu]Lu-PSMA-617: HR, 1.04 per 10 U/L; 95% CI, 1.02–1.07 per 10 U/L; *P* < 0.01 for both groups) (Tables 2 and 3).

**TABLE 2.** ** **Univariable Cox Regression Analysis

	[^177^Lu]Lu-PSMA I&T		[^177^Lu]Lu-PSMA-617	
Variable	HR	*P*	HR	*P*
Gleason score	0.99 (0.76–1.30)	0.97	1.10 (0.77–1.59)	0.59
Age at first cycle (y)	0.99 (0.96–1.02)	0.44	0.97 (0.92–1.01)	0.16
Time from ID to RPT (mo)	1.00 (0.99–1.00)	0.07	1.00 (0.99–1.00)	0.15
eGFR (mL/min), per 10	1.08 (0.93–1.25)	0.32	1.04 (0.89–1.23)	0.68
Leukocytes (per 10³/µL)	0.96 (0.90–1.02)	0.18	0.88 (0.76–1.01)	0.08
Hemoglobin (g/dL)	0.88 (0.75–1.04)	0.14	0.79 (0.68–0.91)	<0.01
Platelets (per 10 × 10³/µL)	1.04 (1.01–1.04)	0.02	1.03 (0.98–1.08)	0.24
CRP (mg/dL)	1.27 (1.14–1.41)	<0.01	1.11 (1.04–1.19)	<0.01
LDH (per 10 U/L)	1.04 (1.02–1.05)	<0.01	1.03 (1.02–1.04)	<0.01
ALP (per 10 U/L)	1.01 (1.01–1.02)	<0.01	1.01 (1.01–1.02)	<0.01
AST (per 10 U/L)	1.01 (1.01–1.02)	<0.01	1.08 (1.04–1.13)	<0.01
GGT (U/L)	1.01 (1.00–1.01)	<0.01	1.01 (1.00–1.01)	<0.01
PSMA-TV (per 100 mL)	1.08 (1.04–1.12)	<0.01	1.08 (1.02–1.13)	<0.01
PSA (per 10 ng/mL)	1.01 (1.00–1.01)	<0.01	1.01 (1.00–1.01)	0.02

ID = initial diagnosis; RPT = radiopharmaceutical therapy; eGFR = estimated glomerular filtration rate; CRP = C-reactive protein; AST = aspartate transaminase; GGT = γ-glutamyltransferase.

Values in parentheses for HR are 95% CI.

**Table 3.** Multivariable Cox Regression Analysis

	[^177^Lu]Lu-PSMA I&T		[^177^Lu]Lu-PSMA-617	
Variable	HR	*P*	HR	*P*
CRP (mg/dL)	1.07 (0.93–1.24)	0.32	1.06 (0.96–1.17)	0.27
LDH (per 10 U/L)	1.03 (1.01–1.05)	<0.01	1.04 (1.02–1.07)	<0.01
ALP (per 10 U/L)	1.01 (0.99–1.02)	0.39	1.00 (0.99–1.02)	0.68
PSMA-TV (per 100 mL)	1.05 (0.99–1.10)	0.11	0.99 (0.91–1.07)	0.79

CRP = C-reactive protein.

Values in parentheses for HR are 95% CI.

### Early PSA Decline and OS

**FIGURE 4.**
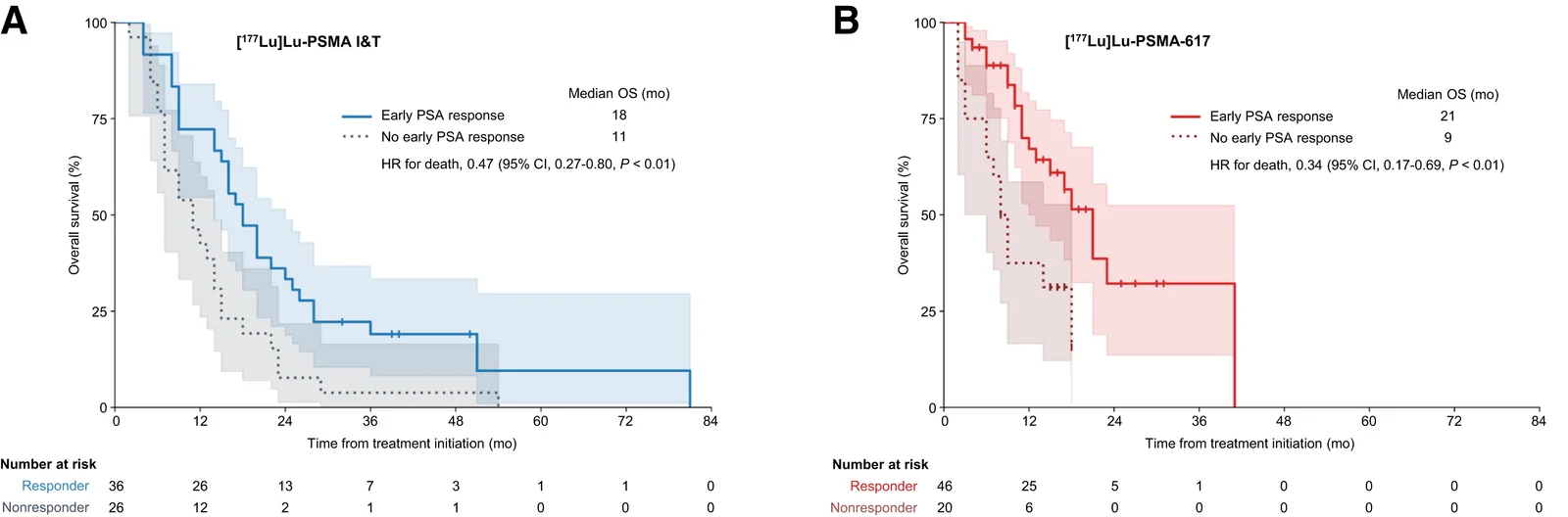
Kaplan–Meier curves for OS from time of treatment initiation, stratified by early PSA response status for [^177^Lu]Lu-PSMA I&T (A) and [^177^Lu]Lu-PSMA-617 (B) cohorts.

Patients demonstrating an early PSA response experienced significantly longer OS compared with nonresponders in both treatment cohorts (Figs. 4A and 4B). In the [^177^Lu]Lu-PSMA I&T group, early responders demonstrated a median OS of 18 mo (range, 14–24 mo) compared with 11 mo (range, 7–14 mo) in nonresponders (HR, 0.47; 95% CI, 0.27–0.80; *P* < 0.01). Similarly, in the [^177^Lu]Lu-PSMA-617 cohort, early responders had a median OS of 21 mo (range, 12 mo–not reached) versus 9 mo (range, 3–18 mo) in nonresponders (HR, 0.34; 95% CI, 0.17–0.69; *P* < 0.01).

### Comparison of Hematologic Toxicity Profiles Between Groups

**FIGURE 5.**
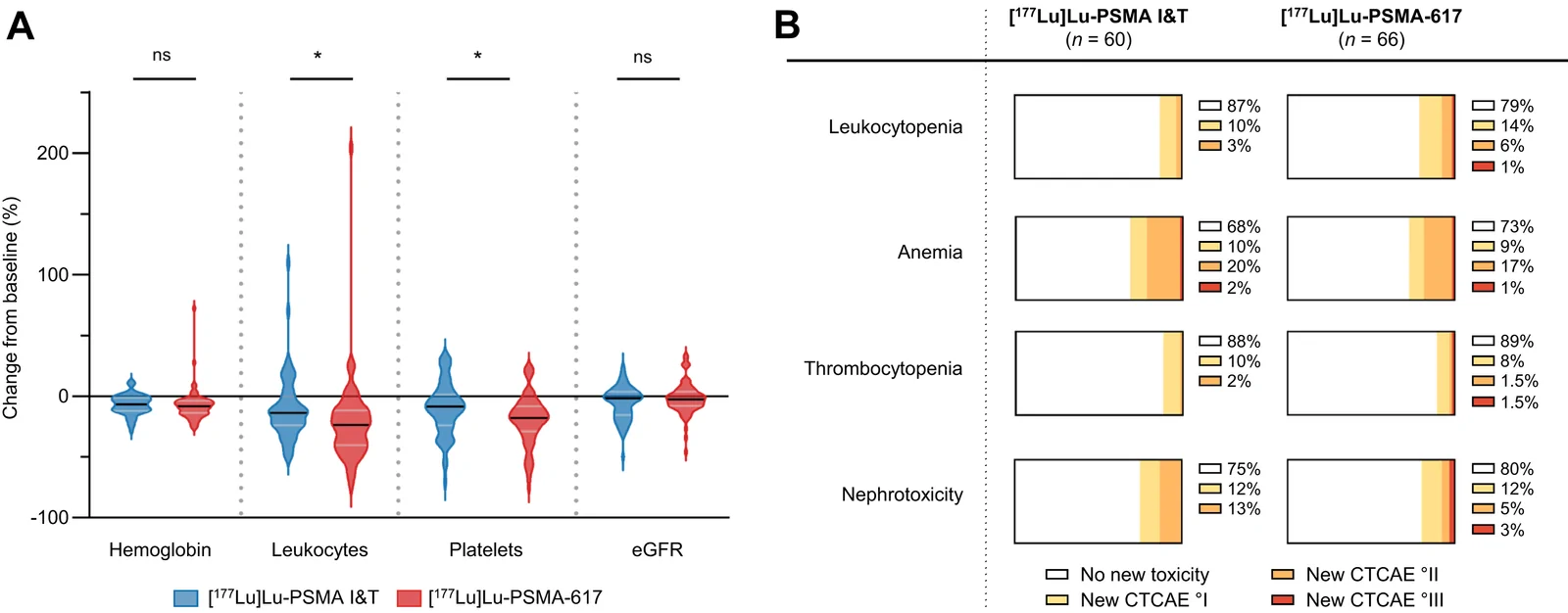
Changes in hematologic and renal function parameters (A) and adverse events classified in accordance with CTCAE version 6.0 (B) from baseline to end of treatment for both cohorts. °I = grade 1; °II = grade 2; °III = grade 3; eGFR = estimated glomerular filtration rate.

Percentage changes in hemoglobin, leukocytes, platelets, and estimated glomerular filtration rate from baseline to end of treatment were analyzed. Follow-up data were available for 60 patients (86%) and 66 patients (94%) in the [^177^Lu]Lu-PSMA I&T and [^177^Lu]Lu-PSMA-617 cohorts, respectively, attributable to early treatment discontinuation in the remaining patients. Hemoglobin and renal function changes did not significantly differ between groups (Supplemental Table 5). In contrast, compared with the [^177^Lu]Lu-PSMA I&T group, the [^177^Lu]Lu-PSMA-617 cohort demonstrated significantly greater declines in median leukocyte count (−23.6% [range, −74.1% to 204%] vs. −13.9% [range, −50.0% to 109%]; *P* < 0.01) and platelet count (−17.9% [range, range, −75.1% to 23.4%] vs. −8.5% [range, −70.3% to 31.3%]; *P* < 0.05) (Fig. 5A).

Predominantly low-grade CTCAE toxicities and no grade 4 events occurred in both treatment groups (Fig. 5B). Anemia was the most frequently observed hematologic toxicity, affecting 19 (32%) of 60 patients in the [^177^Lu]Lu-PSMA I&T cohort and 18 (27%) of 66 patients in the [^177^Lu]Lu-PSMA-617 group, with 1 grade 3 event in each cohort.

Leukocytopenia occurred in 8 patients (13%) treated with [^177^Lu]Lu-PSMA I&T (all low-grade events) and in 14 patients (21%) treated with [^177^Lu]Lu-PSMA-617 (1 grade 3 event). Thrombocytopenia was observed in 7 patients (12%) treated with [^177^Lu]Lu-PSMA I&T (all low-grade events) and 7 patients (11%) in the [^177^Lu]Lu-PSMA-617 cohort (1 grade 3 event). Renal toxicity occurred in 15 patients (25%) receiving [^177^Lu]Lu-PSMA I&T (all grade 1 or 2) and 13 patients (20%) receiving [^177^Lu]Lu-PSMA-617 (2 grade 3 events).

## DISCUSSION

This study compared clinical outcomes between [^177^Lu]Lu-PSMA-617 and [^177^Lu]Lu-PSMA I&T in a propensity score–matched real-world cohort treated with their respective clinical protocols. The main finding was that OS was comparable between treatments despite differences in treatment intensity and biochemical response. Patients receiving [^177^Lu]Lu-PSMA-617 demonstrated superior biochemical response rates and exhibited slightly more pronounced hematologic changes on a quantitative level, whereas CTCAE-graded toxicity remained comparable between cohorts. Importantly, early PSA response emerged as a robust prognostic marker for prolonged OS in both cohorts. Notably, the higher biochemical response rates observed with [^177^Lu]Lu-PSMA-617 did not translate into a corresponding survival benefit at the cohort level. These findings reveal a discrepancy between improved biochemical response and comparable survival outcomes, suggesting that enhanced initial tumor response may not necessarily result in prolonged OS.

The comparable OS seen between [^177^Lu]Lu-PSMA-617 and [^177^Lu]Lu-PSMA I&T cohorts is consistent with our previous matched-pair analysis, in which both compounds administered under identical treatment protocols demonstrated no significant differences in OS or toxicity profiles (5). In the present study, unadjusted survival analysis before matching showed inferior survival in the [^177^Lu]Lu-PSMA I&T cohort, likely reflecting its more adverse baseline characteristics, including higher tumor burden and elevated LDH levels. After propensity score matching, these baseline differences were effectively balanced, and OS was comparable between cohorts. These findings support the interpretation that the differences observed in OS in unadjusted comparisons may be attributable to baseline disease characteristics rather than intrinsic differences in ligand efficacy.

The superior biochemical response rates observed with [^177^Lu]Lu-PSMA-617 should be interpreted in the context of differences in treatment intensity between compounds. Higher treatment intensity has previously been associated with stronger PSA responses in PSMA-targeted RPT (19*–*21), and we hypothesize that the superior biochemical response observed in the [^177^Lu]Lu-PSMA-617 cohort may reflect a higher cumulative radiation delivery rather than an inherent ligand-specific effect. In support of this, Schuchardt et al. demonstrated comparable mean absorbed tumor doses for both agents (4), suggesting that response differences are more attributable to cumulative administered activity than to ligand-specific dosimetric properties. Nevertheless, in the absence of patient-specific dosimetry data, this interpretation remains speculative, given interpatient variability in pharmacokinetics and biodistribution.

The discrepancy between superior biochemical response and comparable OS likely reflects the multifactorial determinants of survival in mCRPC, where disease heterogeneity and the influence of subsequent therapies may attenuate survival differences independent of initial treatment response (22*,*^23^). On an individual level, however, early PSA response was strongly associated with prolonged OS in both cohorts, confirming its value as a prognostic marker, consistent with prior reports (24*–*26).

The observed discrepancy between biochemical response and OS also provides important context for interpreting recent phase 3 trials investigating PSMA-targeted RPTs. The PSMAfore trial demonstrated a pronounced rPFS benefit with 7.4 GBq of [^177^Lu]Lu-PSMA-617 every 6 wk, whereas the SPLASH trial, which used [^177^Lu]Lu-PSMA I&T at longer intervals for fewer cycles, showed a more modest treatment effect (7*,*^11^). Although these differences have raised questions regarding potential ligand-specific efficacy, our findings, together with our previous comparison (5), support the hypothesis that differences in treatment intensity may reflect differences in PSA response and rPFS without necessarily translating into a corresponding OS benefit.

Beyond treatment-related factors, additional prognostic markers of disease biology influenced patient outcomes. In the multivariable analysis, a higher baseline LDH emerged as the only independent predictor for shorter OS across both treatment cohorts, whereas other tumor burden markers (e.g., PSMA-TV, ALP) and inflammatory parameters (e.g., C-reactive protein) showed no prognostic relevance. These findings confirm LDH as a robust and treatment-independent prognostic marker in patients with mCRPC undergoing PSMA RPT, consistent with its established role in multiple oncologic settings (27*–*29).

Regarding toxicity, patients receiving [^177^Lu]Lu-PSMA-617 demonstrated greater declines in platelet and leukocyte counts compared with the [^177^Lu]Lu-PSMA I&T cohort; however, CTCAE-graded hematologic toxicity rates showed no clinically meaningful differences in severe adverse events. A recent meta-analysis reporting comparable bone marrow absorbed doses (30) supports the interpretation that these quantitative differences reflect higher cumulative administered activity rather than ligand-specific bone marrow toxicity and are consistent with prior reports showing acceptable hematologic toxicity across different PSMA RPT regimens (28*,*^31^).

Several limitations of the present study warrant consideration. The retrospective single-center design and use of propensity score matching, while minimizing selection bias, could not fully account for all confounding variables, including temporal changes in supportive care, patient selection practices, and evolving treatment paradigms. Reflecting the real-world setting, neither dose reduction thresholds nor treatment discontinuation criteria were formally standardized but were determined on an individual basis. Furthermore, the shorter follow-up in the [^177^Lu]Lu-PSMA-617 cohort limits statistical power for survival analyses and leaves survival estimates potentially immature. Also, differences in both ligand formulation and treatment protocols between cohorts preclude definitive conclusions regarding ligand-specific effects. Finally, early PSA response was assessed at cycle 2 day 1, corresponding to different absolute time points between cohorts because of differing treatment intervals, which should be considered when interpreting between-group comparisons of early PSA response rates.

## CONCLUSION

In this propensity score–matched real-world analysis, [^177^Lu]Lu-PSMA I&T and [^177^Lu]Lu-PSMA-617 cohorts achieved comparable OS despite differing treatment intensities and biochemical responses. Although [^177^Lu]Lu-PSMA-617 was associated with greater PSA declines, this did not translate into improved survival outcomes. These findings suggest that treatment protocol characteristics, including administered activity and treatment interval, may influence biochemical response, rather than intrinsic differences in ligand efficacy. Prospective randomized studies are warranted to further clarify the relative contributions of ligand properties and treatment intensity.

## DISCLOSURE

This work was supported by a grant from the Interdisciplinary Center of Clinical Research, University Hospital of Wuerzburg, to Natalie Hasenauer (Z-2/96). Andreas Buck has received speaker honoraria from Novartis. No other potential conflict of interest relevant to this article was reported.

## Notes

KEY POINTS **QUESTION:** Do clinical outcomes and safety profiles of [^177^Lu]Lu-PSMA-617 and [^177^Lu]Lu-PSMA I&T differ in patients with mCRPC under real-world conditions? **PERTINENT FINDINGS:** In a propensity score–matched cohort study of 140 patients with mCRPC (70 per group), no statistically significant difference in OS was observed between [^177^Lu]Lu-PSMA-617 (7.4 GBq every 6 weeks, median of 4 cycles) and [^177^Lu]Lu-PSMA I&T (6.0 GBq every 8 weeks, median of 3 cycles), despite higher cumulative administered activity and higher PSA response rates with [^177^Lu]Lu-PSMA-617. Patients receiving [^177^Lu]Lu-PSMA-617 demonstrated greater declines in leukocyte and platelet counts, while CTCAE-graded hematologic toxicity remained similar between cohorts. **IMPLICATIONS FOR PATIENT CARE:** Both compounds appear comparable in effectiveness and toxicity, while observed differences in biochemical response and hematologic parameters likely reflect differing treatment intensities rather than ligand-specific effects, supporting the use of either agent in clinical practice.
